# Power Line Extraction and Tree Risk Detection Based on Airborne LiDAR

**DOI:** 10.3390/s23198233

**Published:** 2023-10-03

**Authors:** Siyuan Xi, Zhaojiang Zhang, Yufen Niu, Huirong Li, Qiang Zhang

**Affiliations:** School of Mining and Geomatics Engineering, Hebei University of Engineering, Handan 056038, China; xsy18513337214@163.com (S.X.);

**Keywords:** power line extraction, safety distance calculation, point cloud, transmission line inspection

## Abstract

Transmission lines are the basis of human production and activities. In order to ensure their safe operation, it is essential to regularly conduct transmission line inspections and identify tree risk in a timely manner. In this paper, a power line extraction and tree risk detection method is proposed. Firstly, the height difference and local dimension feature probability model are used to extract power line points, and then the Cloth Simulation Filter algorithm and neighborhood sharing method are creatively introduced to distinguish conductors and ground wires. Secondly, conductor reconstruction is realized by the approach of the linear–catenary model, and numerous non-risk points are excluded by constructing the tree risk point candidate area centered on the conductor’s reconstruction curve. Finally, the grading strategy for the safety distance calculation is used to detect the tree risk points. The experimental results show that the precision, recall, and F-score of the conductors (ground wires) classification exceed 98.05% (97.98%), 99.00% (99.14%), and 98.58% (98.56%), respectively, which presents a high classification accuracy. The Root-Mean-Square Error, Maximum Error, and Minimum Error of the conductor’s reconstruction are better than 3.67 cm, 7.13 cm, and 2.64 cm, respectively, and the Mean Absolute Error of the safety distance calculation is better than 6.47 cm, proving the effectiveness and rationality of the proposed tree risk points detection method.

## 1. Introduction

As the main carrier and channel for transporting electric energy in the state grid system, the transmission line corridor features a long transmission distance, a high voltage level, large electricity transmission, and small electricity loss. With the growth of society’s demand for electricity resources, the transmission line corridor has been greatly expanded. In order to ensure safe transmission lines for electricity, the relevant departments invest significant manpower and financial resources to inspect transmission lines every year [1,2,3,4]. The traditional manual inspection method has a high operating intensity, a high risk factor, a low inspection efficiency, and poor line coverage. It relies on the subjective judgement of the inspector and has a low reliability of its results, which cannot meet the modern transmission line inspection requirement of accuracy [5,6,7] and may not be able to capture the intricacies acquired by LiDAR. In order to efficiently and accurately obtain the details of the risk points in the transmission line corridor and to promote the construction and development of a smart power grid, the relevant departments of the State Grid Corporation of China are actively exploring intelligent inspection methods of transmission lines with various technological leverages, such as airborne LiDAR and digital photogrammetry.

Airborne LiDAR technology for transmission line inspection makes up for the shortcomings of the traditional manual inspection method in terms of spatial distance measurement accuracy, spatial localization accuracy, and geometric structure measurement efficiency [8,9]; it can realize the digital scanning and multi-dimensional inspection of transmission lines, which provides a brand-new mode and solution for transmission line inspection [10,11,12]. At present, airborne LiDAR transmission line inspection research mainly includes two aspects: point cloud classification and the detection of risk points in transmission line corridors.

Point cloud classification focuses on power line extraction, which is the basis for realizing the automatic detection of the safe distance between power lines and other ground objects [13,14,15,16], and is also the key research direction of many authors. In general, transmission line corridors contain multiple ground–object classes, such as terrain, vegetation, power lines, noise, roadway, and possibly a small number of buildings, which could make power line extraction more difficult. Different scenarios require different strategies to accomplish point cloud classification.

Before the widespread popularization of laser point clouds, many authors conducted power line extraction studies based on 2D images, which are simple and easy to implement [17]. These methods often use the point-line duality of the Hough transform [18], that is, any portion of a line segment on a straight line corresponds to the same point in the parameter space, and each straight line corresponds to a separate point in the parameter space to represent it. As a result, it is possible to detect lineaments from a two-dimensional plane. Yu et al. [19] extracted power lines from a two-dimensional projection plane with the Hough transform method based on already-classified ground points and vegetation points. Guan et al. [20] also used the Hough transform to extract the power line points in the 2D projection plane and eliminated shorter linear feature disturbances by constraining the straight-line lengths. However, the power line extraction method based on a two-dimensional projection plane cannot effectively distinguish the vertically distributed multilayer conductors of transmission lines and has a poor accuracy of classification.

With the popularization of laser technology and the in-depth study of algorithms, power line extraction based on three-dimensional (3D) space has become mainstream, mainly categorized into rule-based extraction, machine learning (ML), and deep learning (DL). In general, the typical rule-based classification appeared first, containing a series of procedures and rules. For example, ground points are first extracted from the original point cloud to generate the Digital Terrain Model (DTM), and then feature differences and a clustering algorithm are used to distinguish power lines from other ground objects, especially vegetation [21,22]. Finally, the clustering algorithm or region growing algorithm is used to refine the power line extraction results. Rule-based methods heavily focus on the sequential logic of the extraction of different objects and must fully utilize the obvious differences between power lines and ground objects to achieve a good classification accuracy, such as the density, height, and morphometrics. Huang et al. [23] extracted a power line according to the different height distributions of the power line, pylon, and ground object and then refined the result through grid-based region growing. McLaughlin et al. [24] divided a point cloud into a number of ellipsoids, separately calculated the covariance matrix and eigenvalues of the chunked point cloud, and then classified the points with larger eigenvalues in only one direction into the power line category according to the linear features of power lines. Liu et al. [25] used the clustering algorithm and region growth method to realize the segmentation and extraction of a single transmission line. However, these methods are likely to result in object fragmentation, which may impact classification accuracy. Multi-scale analysis can solve this problem, but it requires several calculations and takes some time [26].

Supervised ML algorithms use training data that have been manually or automatically classified as a reference and make predictive classifications [27]. Unsupervised ML algorithms do not require training data and classify the data based on the similarity between the samples, which can be classified into four families: classification trees such as random forest (RF), grouping and separation methods such as support vector machine (SVM) and k-nearest neighbor (KNN), and rule application methods such as convolutional neural networks (CNNs) [28]. ML classification methods rely heavily on point cloud index structure organization, feature extraction, and construction and classifier selection [29,30,31,32,33,34]. For point clouds in a transmission line corridor, the sample sets should cover a wide range of power line types and tower types, the feature sets should be explicit and efficient, and the classifiers should be appropriate [35,36].

The wide application of DL in 2D image classification, target detection, and other remote sensing data processing provides a new idea and direction for 3D laser point cloud classification, which has the ability to express the deep information of the features and has high classification accuracy for disordered and unstructured point clouds [37,38,39]. Yang et al. [40] utilized a 3D convolutional neural network and combined it with the KITTI dataset for training and achieved power line point cloud extraction, which removed the dependence on 2D images. Qi et al. [41] proposed VoxelNet based on a 2D CNN, which divides the point cloud into voxels of the same size and achieves end-to-end detection by learning the features of each voxel. Zhang et al. [42] proposed ShellConv, a convolution operator that can be used to directly process point cloud data in 3D space, and designed a ShellNet based on it, acting directly on 3D point clouds like a 2D convolution operation to extract local domain information. Currently, deep learning for point cloud classification faces two main challenges: one is the irregularity and disorder of the point cloud, leading to the existing neural network and convolution not being adapted; another is that the local and global features of the point cloud are not described comprehensively enough.

For transmission line point clouds with complex situations and large scenarios, a large number of point cloud features are inevitably needed to obtain better classification results, thus consuming a lot of time. Therefore, ML methods and DL methods cannot achieve both classification accuracy and efficiency when facing the point clouds of complex scenes, and the universality still needs to be improved.

Tree risk is a safety issue caused by trees being too close to a conductor. This is one of the most common risks of transmission lines, causing accidents with greater destructiveness and higher incidence, and even a single tree may make contact with the conductor and cause a wildfire [43]. From a utility business perspective, all trees capable of growing into or on failure striking a power line are not only a legal liability due to human safety and property concerns but are also a financial liability [44]. Thus, the timely identification of tree risk points will greatly facilitate risk management in respect of transmission lines, reducing outages and increasing the resiliency of transmission lines [45]. Tree risk point detection is mainly based on a point cloud to determine the distance between the tree and the conductor and compare it with the safety distance stipulated by the relevant departments; tree points that do not meet the safety distance requirements are regarded as tree risk points. Chen et al. [10] proposed a piecewise clearance calculation method that converts the point-to-catenary curve distance measurements to a minimal distance calculation based on differential geometry; this is used to calculate the distance between the power line and the tree. Dihkan et al. [46] segmented spaces according to a certain step size and used each space as a basic unit for risk detection. This method is sensitive to the selection of step size; too small a step size will increase the amount of computation and reduce the computational efficiency, and too large a step size will lead to the omission of risk points. Chen et al. [47] segmented the vector of a power line according to a certain distance, then calculated the safety distance between the power line and the tree in each profile and compared it with the safety distance of the neighboring profiles, taking the minimum value as the safety distance. Currently, there is little research on automated and intelligent detection methods of tree risk based on point clouds, and the existing research is not extensive enough. The reasonableness and effectiveness of risk detection methods need to be further improved.

Despite the fact that the above recently proposed methods have significantly improved the performance of power line extraction and tree risk detection, the main challenges still need to be addressed. These include the following:

(1) Although the power line extraction methods based on linear features are mainstream, they rely heavily on geometric constraints and preset parameters, ignore the spatial distribution features of transmission lines, and do not further distinguish differences between aerial ground wires and conductors, which is not conducive to the efficient inspection of transmission lines.
(2) Automated and intelligent tree risk detection methods based on 3D point clouds focus on how to use different heights of spatial segmentation methods, different neighborhood space determination methods, and different point cloud index structures to achieve the rapid point-to-point or point-to-line safety distance calculation. However, they ignore the obvious spatial distribution features of transmission lines and the actual situation, in which the number of tree risk points is small, resulting in a large number of non-essential safety distance calculations and judgments.

To overcome the above problems, we propose a new power line extraction and tree risk detection method that makes full use of spatial distribution features. The main contributions of the proposed method are the following three aspects:

(1) The local dimensional feature probability model of a point cloud under the restriction of minimum information entropy is proposed to realize the accurate extraction of power lines, and the method still has good applicability for complex scenarios.
(2) The cloth simulation filtering (CSF) algorithm [48] and region growth method based on the neighborhood sharing degree are used to achieve an accurate distinction between ground wires and conductors.
(3) The candidate area of tree risk points centered on the conductor reconstruction curve based on a catenary-linear equation is constructed, and a safety distance grading calculation strategy is proposed to realize the accurate detection of tree risk points.

## 2. Methods

In this section, the logic of the proposed approach is described in detail. Firstly, the overall architecture is outlined. Then, the proposed methods are elaborated on separately.

### 2.1. Overall Architecture

**Figure 1:**
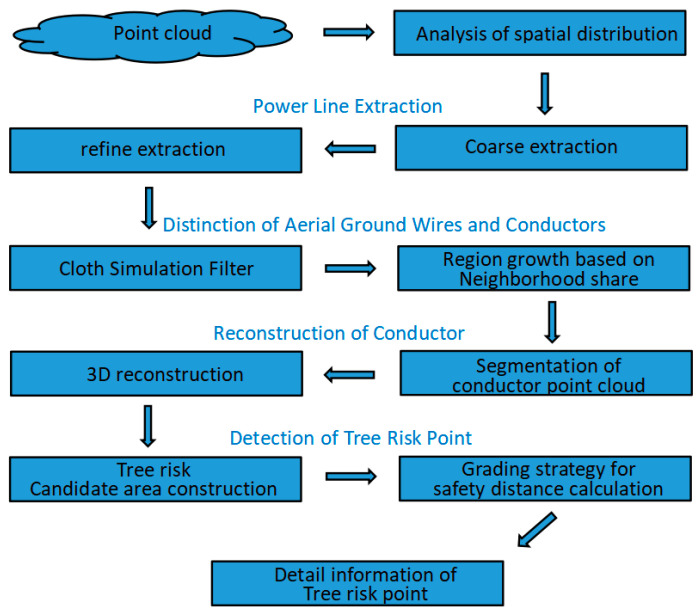
Overview of the method.

As shown in Figure 1, the proposed method includes five steps: analysis of spatial distribution features of transmission lines, power line extraction, distinction between conductors and ground wires, 3D reconstruction of the conductor, and tree risk detection.

### 2.2. Spatial Distribution Feature Analysis of Transmission Line

Transmission line corridors are generally composed of ground wires, conductors, pylons, and other components. The spatial distributions and morphological features of ground wires are very similar to conductors. Pylons connect the power lines and run through the entire transmission line corridors. Transmission lines are typical human-made objects with distinct spatial features.

**Figure 2:**
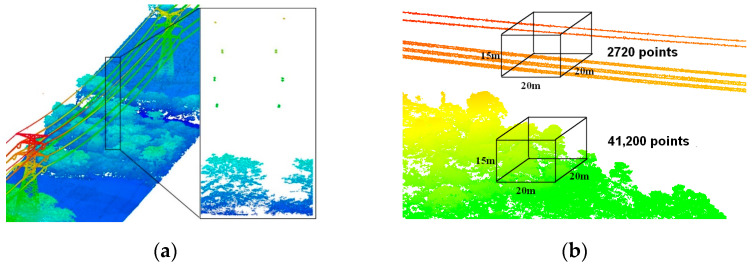
Spatial distribution of transmission lines: (**a**) features of vertical spatial distribution of transmission lines; (**b**) differences in the number of vegetation and power line points in the same area. Different colors represent different elevations.

(1) As shown in Figure 2a, power lines are suspended from the tower in the form of a catenary and the ground wires are above the conductors. The distances between different power lines are kept fixed and distributed approximately in parallel. A single power line is closely connected end to end in the horizontal direction with obvious linear features. However, the vertical distribution of the power line is extremely discontinuous.
(2) Within the same size area, there are significantly more vegetation points than power line points, which means that when counting some of the indicators related to the point coordinates or numbers, the vegetation points are given more weight. Randomized comparisons of power line point density and vegetation point density are both applicable, as shown in Figure 2b.

### 2.3. Power Line Extraction

Airborne radar detects objects blindly, and multiple object point clouds are mixed together, which is not conducive for power line extraction. Among these, vegetation points and ground points are the most common, and power line points are rare, so power line extraction is divided into two steps: power line coarse extraction and refined extraction.

#### 2.3.1. Coarse Extraction of Power Lines Based on Height Difference

**Figure 3:**
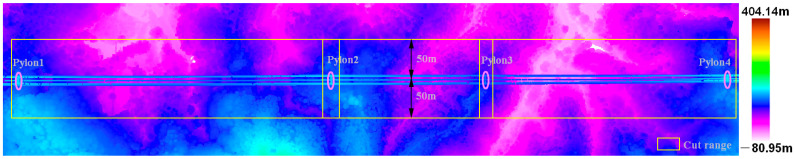
Point cloud cutting. The pink circles represent the location of the pole tower and the width of the arrows represents the width of the cut range.

**Figure 4:**
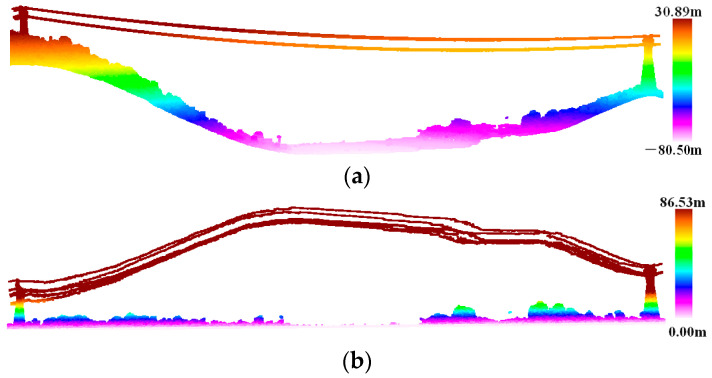
Point cloud elevation normalization: (**a**) original point cloud elevation display via elevation rendering method; (**b**) normalized point cloud elevation display via elevation rendering method.

**Figure 5:**
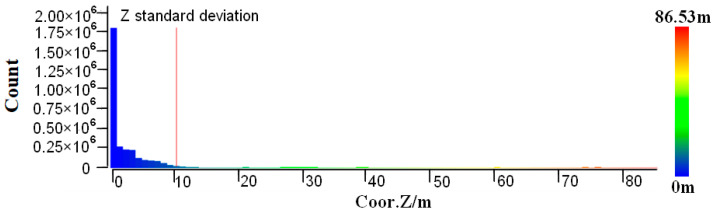
Number of points in different elevation ladders.

In order to reduce the number of point clouds that clearly do not belong to the power line category during the power line extraction process and improve the efficiency and accuracy of power line extraction, height differences between vegetation and the power line are used as a judgment condition by which to roughly extract the candidate points of the power line. The algorithm is described as follows:

(1) In order to ensure the integrity of the transmission line, the original point cloud width is much larger than the transmission line corridor width. Therefore, the point cloud needs to be clipped according to the coordinates of the pylons already known by the State Grid Corporation of China and the specified corridor width, which is generally 100 m. The original point cloud is divided into multiple point clouds that are end to end, each containing two adjacent towers, power lines, and other features, as shown in Figure 3.
(2) In order to eliminate the influence of terrain undulation, facilitate subsequent point elevation statistics, and distinguish ground and non-ground points, an improved progressive TIN densification filtering algorithm is used to obtain the terrain of the transmission line corridor [49]; then, the point cloud is elevation normalized based on the ground point, as shown in Figure 4.
(3) Starting from the ground, the number of non-ground points corresponding to different elevation ladders is counted with 1 m as the step length. The reason for the 1 m step length is that there is more low vegetation and less medium and high vegetation, and vegetation that is 0–1 m above ground level is considered low vegetation. As shown in Figure 5, the elevation of the points is mainly within 10 m; this predominantly comprises low vegetation close to the ground, with the most points within 0–1 m. However, there are very few points with an elevation greater than 10 m; these are mainly power line points and pylon points.
(4) The Z standard deviation [50] of the non-ground points is calculated using Equation (1).
  (1) $$S\left(Z\right)=\sqrt{\frac{\sum_{i=1}^{n}{\left(Z_{i}-\bar{Z}\right)}^{2}}{n}}$$

Here, S(Z) denotes the Z standard deviation; $Z_{i}$ and $\bar{Z}$ represent the elevation of each point and average elevation values of the point cloud, respectively; and *n* is the number of points. Vegetation points are heavily weighted and power line points are lightly weighted; S(Z) is closer to the vegetation point elevations. As shown in Figure 5, the Z standard deviation is almost the same with the elevation corresponding to the abrupt change in the number of points.

**Figure 6:**
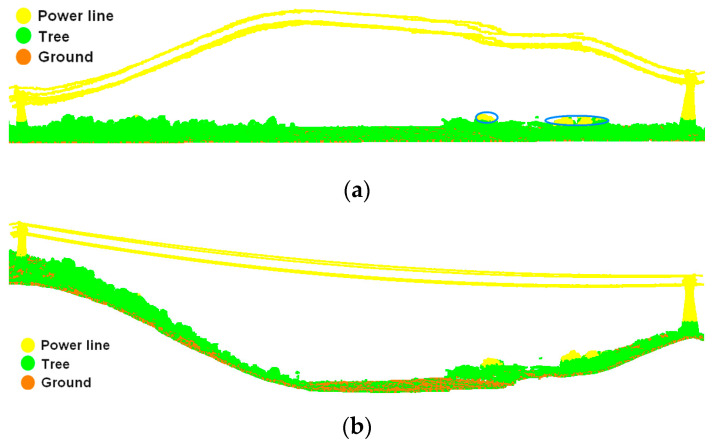
Coarse extraction of power line: (**a**) coarse extraction effect of normalized point clouds; (**b**) coarse extraction of inverse normalized point cloud.

(5) Points with elevations greater than the Z standard deviation are classified as power line candidate points, and converse situations are classified as vegetation points. The effect of power line coarse extraction is shown in Figure 6; all power line candidate points have been extracted completely and accurately, while a very small number of vegetation canopy points have also been categorized as power lines, such as the points in the blue circle in Figure 6a. This misclassification will be improved in the subsequent power line refined extraction.
(6) Elevation-normalized point clouds are denormalized based on ground points to restore the original elevation of points, as shown in Figure 6b.

#### 2.3.2. Refined Extraction of Power Lines Based on Local Dimensional Features Probability Model

The local dimensional features of power lines, pylons, and tree canopies vary greatly. The one-dimensional linear features of power lines are extremely obvious, pylons are assembled with metal facets and have obvious two-dimensional planar features, and tree canopies grow freely in all directions and present irregular three-dimensional spherical features. Therefore, power line refined extraction is transformed into a point cloud local dimension feature analysis problem. Although there is no clear geometric topological relationship between points, the local dimensional features of the point cloud can be described by the eigenvalues and eigenvectors.

**Figure 7:**
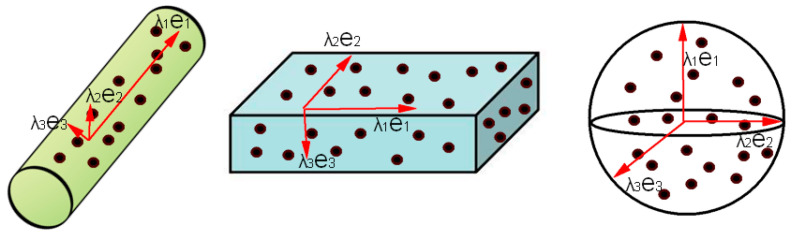
The relationship between eigenvalue size and the local dimension features of the point cloud. The black dots represent the point cloud and the arrows indicate the pointing of the feature vectors.

The eigenvalues ($\lambda_{1},\lambda_{2},\lambda_{3}$) and eigenvectors ($E_{1},E_{2},E_{3}$) can be obtained by constructing the neighborhood covariance matrix via principal component analysis (PCA) [51]. The size of the eigenvalue represents the dispersion of neighboring points in a certain direction; this means that when the eigenvalue is larger, the neighborhood points are more concentrated in the direction of the corresponding eigenvector [52]. Thus, the eigenvalues can reflect the local dimensional features of the point cloud initially, as shown in Figure 7.

- When $\lambda_{1}\gg \lambda_{2}\approx \lambda_{3}$, the local feature of the point cloud is one-dimensional linear;
- When $\lambda_{1}\approx \lambda_{2}\gg \lambda_{3}$, the local feature of the point cloud is two-dimensional planar;
- When $\lambda_{1}\approx \lambda_{2}\approx \lambda_{3}$, the local feature of the point cloud is three-dimensional spherical.

**Figure 8:**
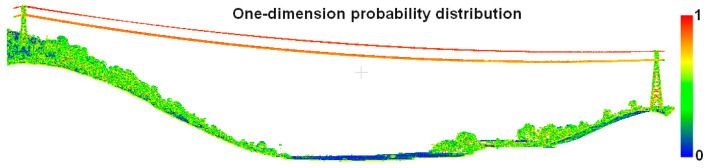
One-dimension probability of point cloud.

In order to describe the point cloud dimension accurately, we introduce a point cloud dimension feature probability model based on Equation (2), which is the ratio between the eigenvalue differences and the eigenvalues, and is similar to the method created by Guan et al. [52]. Using this method, the size of the ratio represents the degree of similarity of the eigenvalues, which is clearer than only comparing the size of the eigenvalues. The calculation formula is as follows:

(2) $$\left\{ \begin{array}{l} L_{\lambda }=\frac{\lambda_{1}-\lambda_{2}}{\lambda_{1}}\\ P_{\lambda }=\frac{\lambda_{2}-\lambda_{3}}{\lambda_{1}}\\ S_{\lambda }=\frac{\lambda_{3}}{\lambda_{1}} \end{array}\right.$$

where $L_{\lambda },\;P_{\lambda }$, and $S_{\lambda }$ denote the probability that the point cloud belongs to one-dimensional, two-dimensional, and three-dimensional features, respectively, and can be used to describe the spatial distribution of the point cloud within the local neighborhood. The sum of these variables is equal to 1. The points with the highest probability of one-dimensional features are classified as power line points to realize power line fine extraction. Figure 8 demonstrates the one-dimensional feature probability distribution of the point cloud, and it can be clearly seen that the power line point cloud has the largest one-dimensional probability, which is close to one. In addition, there are very few red points in the pylon and vegetation, which is caused by incorrect eigenvalues. It has been shown that the neighborhood size has a large impact on estimation of the eigenvalue and eigenvector [53], so an appropriate neighborhood radius is crucial for refined extraction of the power line.

In order to find the optimal neighborhood radius and accurately portray the local dimensional features of the point cloud, the entropy function [54] is introduced to calculate the local information entropy of the point cloud under different neighborhood sizes, as shown in Equation (3). According to the theory of minimum information entropy [54], the entropy represents the degree of chaos, and smaller entropy values represent a more unified system. Therefore, when the information entropy is the smallest, this means that the local point cloud has a high degree of consistency, which helps to solve the eigenvalue and the determination of the characteristic probability of the local dimensions.

(3) $$\left\{ \begin{array}{l} H_{f_{i}}=-\left(L_{\lambda_{i}}\log_{e}\left(L_{\lambda_{i}}\right)+P_{\lambda_{i}}\log_{e}\left(P_{\lambda_{i}}\right)+S_{\lambda_{i}}\log_{e}\left(S_{\lambda_{i}}\right)\right)\\ R_{\mathrm{excel}}=\mathrm{argmin}\left(H_{f_{i}}\right) \end{array}\right.$$

where $H_{f_{i}}$ denotes the information entropy of the *i*th point; $R_{\mathrm{excel}}$ represents the neighborhood radius corresponding to the minimum information entropy of the *i*th point; and $L_{\lambda_{i}},\;P_{\lambda_{i}}$, and $S_{\lambda_{i}}$ denote the probability that the *i*th point belongs to one-dimensional, two-dimensional, and three-dimensional features, respectively.

To show more clearly the feature probabilities and information entropy corresponding to different neighborhood radii, Table 1 takes a point of the power line as an example for calculation. By analyzing Equation (3) and Table 1, it can be seen that the smaller the information entropy value of the point, the higher the probability of a certain dimension. Therefore, it is reasonable to use the minimum value of the information entropy to determine the optimal neighborhood radius of each point.

**Figure 9:**
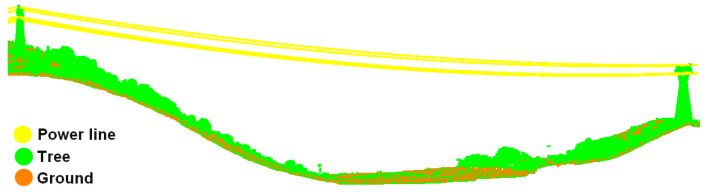
Refined extraction of power lines.

In order to avoid the possibility of vegetation and tower edges presenting linear features affecting the power line classification accuracy, Table 2 lists the standard deviation (SD), and the maximum and minimum values of the angles between the principal eigenvectors and horizontal plane for each classification of points. It is clear to see that the standard deviation of the angle of the power line points is almost equal to zero, and the maximum angle is within 5 degrees. Therefore, 5 degrees is used as a constraint by which to improve the classification accuracy. The refined extraction effect of the power line is shown in Figure 9.

### 2.4. Distinction between Aerial Ground Wires and Conductors

Due to the extremely similar morphological features of conductors and ground wires, existing studies usually regard them as being in the same category [13,14,15,16,55]. However, the relevant departments of the State Grid Corporation of China make clear distinctions about both, so it is necessary to explore the automatic differentiation methods of conductors and ground wires.

#### 2.4.1. Coarse Extraction of Aerial Ground Wires Based on CSF

**Figure 10:**
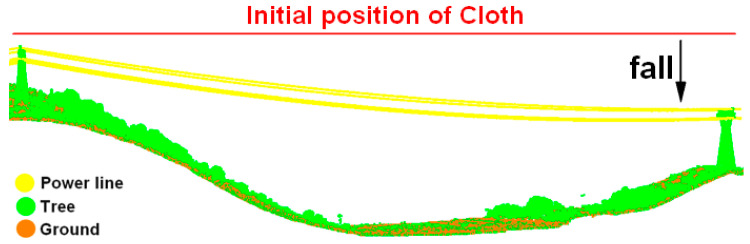
Schematic of CSF.

CSF is a simulation of a physical process assuming that a piece of cloth descends from above a point cloud slowly until it comes to stop [48], as shown in Figure 10. Power line points are extracted based on the vertical distance between the power line and the cloth. If the vertical distance is less than the threshold, the power line point cloud is categorized as ground wires; otherwise, it is categorized as conductors.

**Figure 11:**
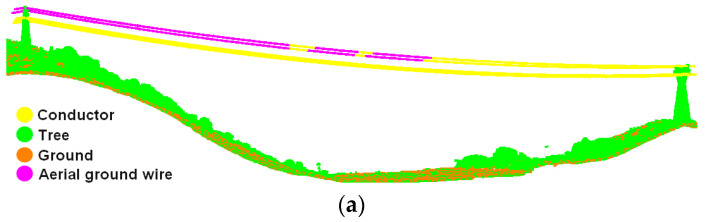

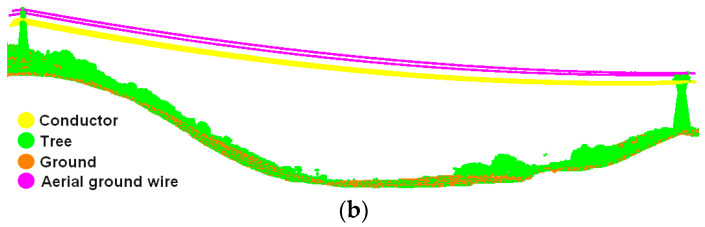
Distinction between conductors and ground wires: (**a**) coarse extraction of ground wires based on CSF; (**b**) refined extraction of ground wires based on neighborhood sharing.

In order to avoid the cloth particle falling into the gaps between the power lines and leading to errors in extracting ground wires, the cloth should be hard enough and the cloth resolution should be moderate. Due to the fact that power lines take the form of catenaries and the heights of pylons are varied, CSF can only identify partial ground wires, as shown in Figure 11a.

#### 2.4.2. Refined Clustering of Ground Wires Based on Degree of Neighborhood Sharing

**Figure 12:**
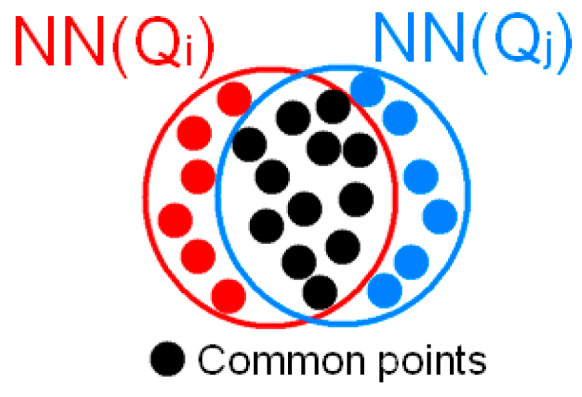
Diagram of neighborhood sharing. The red dots belong to NN(Q*_i_*), the blue dots belong to NN(Q*_j_*), and the black dots are common points.

In this section, a region growth method based on neighborhood sharing is proposed, which determines whether two points can be clustered into one category by comparing the sharing degree of the neighboring point sets NN(Q*_i_*) and NN(Q*_j_*). As shown in Figure 12, the more common points owned by the two neighboring point sets indicate a higher sharing degree and a higher possibility of clustering the two points into the same category. Compared with Euclidean clustering [56] or normal vector angle-based region growth [57], this method directly uses the number of common points to determine whether two points can be clustered into the same category, avoiding the calculation of distance or angle. In addition, this method is more compatible with the spatial distribution characteristics of conductors and ground wires. The effect of ground wire clustering is shown in Figure 11b.

### 2.5. 3D Reconstruction of Conductors

The classified conductor point cloud contains multiple conductors and cannot be used for reconstruction directly, so single conductor segmentation is required. On the vertical plane where the conductors are located, the point cloud shows significant aggregation and there are certain spatial distances and intervals between different conductors, so the density-based spatial clustering of application with noise (DBSCAN) method can be utilized to decompose multiple conductors into a single conductor [58].

**Figure 13:**
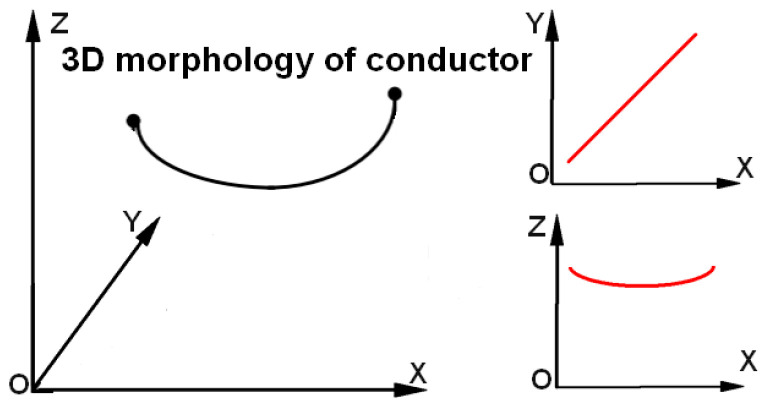
Conductor morphology analysis. In the X-O-Y projection plane, the red line is a straight line, and in the X-O-Z projection plane, the red line is a catenary.

In order to describe the spatial features of a single conductor accurately, it is decomposed in different projection planes. As shown in Figure 13, a linear equation is chosen to describe the conductor morphology in the X-O-Y projection plane and a catenary equation is chosen to describe the conductor morphology in the X-O-Z projection plane.

#### 2.5.1. Linear Equation

To introduce the angle of the line and the perpendicular distance from the origin to the line, the point-normal equation is used to describe the conductor morphology in the X-O-Y plane this is shown in Equation (4).

(4) T=x×cosθ+y×sinθ

Here, *T* represents the length of a vertical segment that is perpendicular to the linear equations through the origin; θ denotes the angle between the vertical segment and the X-axis, specifying the counterclockwise direction as the positive direction. The parameters of linear equations can be determined using the least squares method.

In order to determine the location of the endpoints of the line, the projection range factor μ is defined through trigonometric functions, which are shown in Equation (5) and are used as a link between the linear equation and catenary equation.

(5) $$\left\{ \begin{array}{l} \mu =\frac{x_{i}^{\prime }-x_{\mathrm{fp}}}{-\sin\theta }\;\mathrm{if}\;\sin\theta \geq \cos\theta \\ \mu =\frac{y_{i}^{\prime }-y_{\mathrm{fp}}}{-\cos\theta }\;\mathrm{if}\;\sin\theta <\cos\theta \end{array}\right.$$

Here, ($x_{i}^{\prime },y_{i}^{\prime }$) are the projection coordinates obtained by the point cloud in the X-O-Y plane; ($x_{\mathrm{fp}},y_{\mathrm{fp}}$) are the coordinates of the intersection of the linear and vertical segment. The maximum and minimum values of μ are the two endpoints of the line.

#### 2.5.2. Catenary Equation

In the X-O-Z projection plane, the power line is in the form of a catenary. Therefore, we introduce the catenary equation from ref. [2] to fit the power line. The formula is as follows:

(6) $$a*\cos h\left(a\mu +b\right)+c=\frac{a\left(e^{a\mu +b}+e^{-a\mu -b}\right)}{2}+c$$

where *a, b*, and *c* are the parameters of the catenary equation. In order to calculate the parameters more conveniently, we simplify the catenary using Taylor’s formula [59]; the higher the degree of the polynomial, the more accurate the equation fitting result will be. Therefore, we simplify the catenary to a fourth-degree polynomial; the formula can be expressed as

(7) $$Z=a+a\frac{{\left(a\mu +b\right)}^{2}}{2}+a\frac{{\left(a\mu +b\right)}^{4}}{24}+c$$

where *Z* denotes the fitted elevation of the conductor using a polynomial. The optimal parameters of Equation (7) are determined using the least-squares method [60], which is mathematically expressed as

(8) $$F\left(a,b,c\right)={\sum_{i=1}^{n}\left(a+a\frac{{\left(a\mu_{i}+b\right)}^{2}}{2}+a\frac{{\left(a\mu_{i}+b\right)}^{4}}{24}+c-Z_{i}\right)}^{2}$$

The partial derivatives of *a*, *b*, and *c* in Equation (8) are calculated to obtain Equation (9).

(9) $$\left\{ \begin{array}{l} \frac{\partial F}{\partial a}=2\sum_{i=1}^{n}\left(a+a\frac{{\left(a\mu_{i}+b\right)}^{2}}{2}+a\frac{{\left(a\mu_{i}+b\right)}^{4}}{24}+c-Z_{i}\right)*\frac{\partial \left(Z-Z_{i}\right)}{\partial a}\\ \frac{\partial F}{\partial b}=2\sum_{i=1}^{n}\left(a+a\frac{{\left(a\mu_{i}+b\right)}^{2}}{2}+a\frac{{\left(a\mu_{i}+b\right)}^{4}}{24}+c-Z_{i}\right)*\frac{\partial \left(Z-Z_{i}\right)}{\partial b}\\ \frac{\partial F}{\partial c}=2\sum_{i=1}^{n}\left(a+a\frac{{\left(a\mu_{i}+b\right)}^{2}}{2}+a\frac{{\left(a\mu_{i}+b\right)}^{4}}{24}+c-Z_{i}\right) \end{array}\right.$$

**Figure 14:**
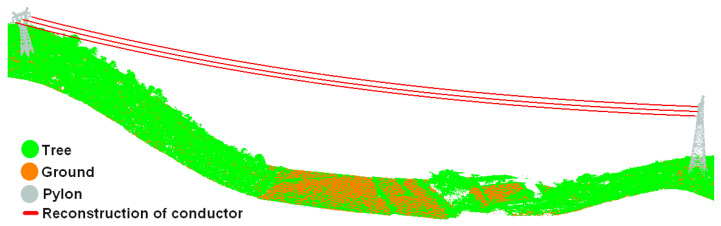
3D reconstruction of conductors. In this paper, we use existing methods [11] combined with manual classification to extract the point clouds of pylons.

Here, *n* is the number of point clouds participating in the conductor fitting; $Z_{i}$ and $\mu_{i}$ are the elevation and projection range of the *i*th conductor point; and *Z* denotes the fitted elevation calculated using Equation (7). The optimal parameters of Equation (7) can be obtained by taking all of the above partial derivatives to zero. The effect of 3D reconstruction of the conductors is shown in Figure 14.

### 2.6. Tree Risk Detection

In general, only a small amount of overgrown vegetation will pose a threat to the safe operation of transmission lines. However, there is substantial vegetation in the transmission line corridor, and traversing each point to calculate the safety distance will result in redundant non-essential calculations. We therefore propose a strategy to exclude a large number of non-risk points by constructing the tree risk candidate area and then detecting tree risk points before and after the process, with grid and independent points as the minimum basic configuration.

#### 2.6.1. Construction of Tree Risk Candidate Area

Typically, the vast majority of trees do not interfere with the safe running of transmission lines. Therefore, we construct the candidate area of tree risk to exclude most of the non-risk points and significantly reduce the safety distance computation based on point clouds.

**Figure 15:**
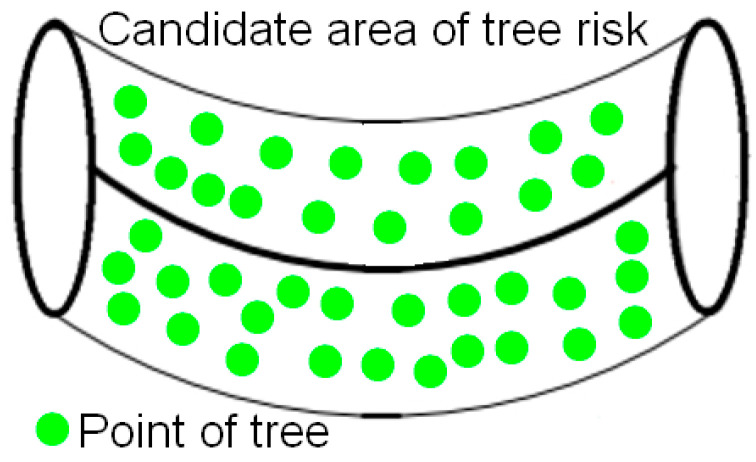
Construction of tree risk candidate area.

The height of the conductor is not consistent at each location, so the point cloud involved in tree risk detection should be within a cylindrically similar area that is centered on the reconstruction results for the conductors, as shown in Figure 15. In order to avoid the missed identification of tree risk due to a radius that is too small, the radius of the cylindrically similar area should be larger than the maximum safety distance specified for the corresponding voltage level.

#### 2.6.2. Rough Calculation of Safety Distance Based on Grid

In order to further reduce the redundant calculation caused by non-risk points in the candidate area of tree risk, a 3D grid is used as the basic unit to simplify the morphology and number of point clouds in the candidate area. Assuming that the sequence number of the grid cell containing the conductor is *i* and the grid number containing trees is *j*, the spatial distance between both is calculated using the following formula:

(10) $$D_{ij}=\sqrt{{\left(x_{i}-x_{j}\right)}^{2}+{\left(y_{i}-y_{j}\right)}^{2}+{\left(z_{i}-z_{j}\right)}^{2}}$$

where ($x_{i},y_{i},z_{i}$) are the center coordinates of the *i*th grid cell and ($x_{j},y_{j},z_{j}$) are the center coordinates of the *j*th grid cell. Rough calculation of the safe distance is only used for grid cells that are in adjacent rows or in the same row.

There is an extreme case in which conductor points and tree points happen to be located at the inner vertex of the two grid cells, so the actual distance should be the difference between $D_{ij}$ and the diagonal length. The formula is as follows:

(11) $$D_{\mathrm{actual}}=D_{ij}-\sqrt{3}l_{0}$$

where $l_{0}$ is the diagonal length. $D_{\mathrm{actual}}$ is compared with the specified safety distance $D_{\mathrm{thre}}$ of the transmission line. If $D_{\mathrm{actual}}>D_{\mathrm{thre}}$, the point clouds of the trees within the grid are labeled as non-hazardous attributes and are no longer involved in the subsequent safety distance calculation; otherwise, they are marked as pending the judgment state and participate in the subsequent safe distance calculation.

#### 2.6.3. Accurate Calculation of Point-to-Point Safety Distance

**Figure 16:**
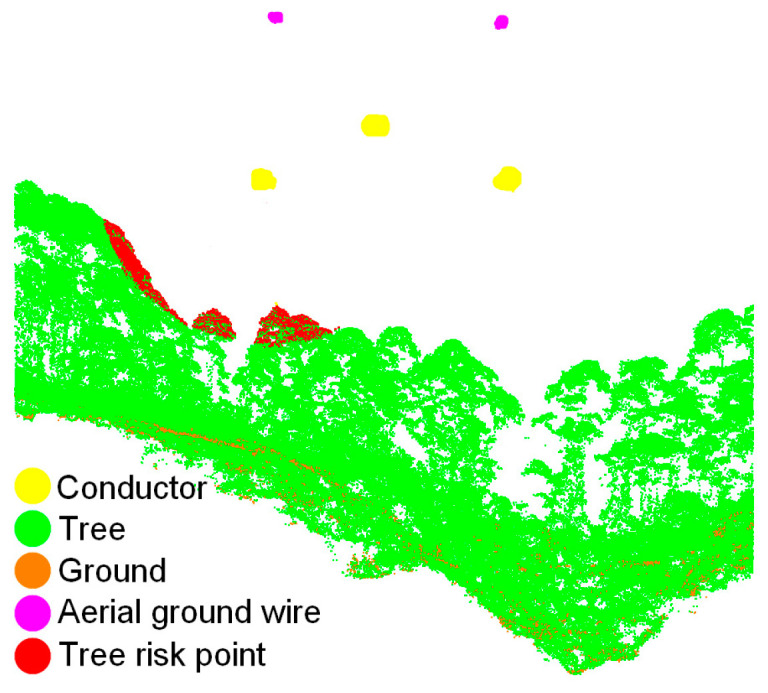
Detection results for tree risk points.

Among the point clouds of trees that need further judgment, there are many risk points and few non-risk points. In this case, the safety distance calculation based on point-to-point safety distance not only ensures the correctness of the identification of tree risk points, but also avoids a large number of invalid calculations and redundant calculations. The judgment conditions of tree risk points are as follows:

(12) $$\left\{ \begin{array}{ll} 0.5m<D_{\mathrm{ptp}}\leq D_{\mathrm{thre}} & \;\mathrm{Risk}\\ D_{\mathrm{ptp}}>D_{\mathrm{thre}} & \;\mathrm{Non}-\mathrm{risk} \end{array}\right.$$

where $D_{\mathrm{ptp}}$ represents the point-to-point clearance distance. If the clearance distance between the conductor and the tree is between 0.5 m and $D_{\mathrm{thre}}$, the tree points may threaten the safe running of the transmission line, so they are judged as tree risk points. The purpose of setting the minimum threshold of the safety distance is to avoid misidentification of tree risk points due to misclassification of point clouds. The detection results for the tree risk points are shown in Figure 16.

## 3. Experiments and Discussion

Three groups of transmission line point clouds with different voltage levels were selected to verify the practicality and accuracy of the method proposed in this paper, which mainly comprises three parts: classification accuracy of ground wires and conductors, spatial position accuracy of conductor 3D reconstruction, and accuracy of tree risk point detection.

### 3.1. Datasets

The experimental data were acquired by a long-range airborne LiDAR system named VUX-240 from Rigel, Austria, and the flight platform is a fixed-wing UAV flying at an altitude from 200 m to 220 m. The details of the datasets are listed in Table 3. Datasets were acquired in different regions, and the voltages of the transmission lines are 110 kV, 220 kV, and 500 kV. The corridors cover common terrain such as flat ground, hills, and mountains, as well as contain a large number of trees. The length of the 110 kV transmission line is 376 m with six conductors, the length of the 220 kV transmission line is 227 m with six conductors, and the length of the 500 kV transmission line is 591 m with three conductors. In order to reduce the amount of computation, the experimental data were sampled prior to data classification.

### 3.2. Evaluation Methods

This section defines the methods used for calculating the accuracy of point cloud classification and evaluating the rationality of tree risk point detection, which mainly involves conductor (ground) classification accuracy estimation, conductor 3D reconstruction position accuracy assessment, and tree risk point detection accuracy assessment.

#### 3.2.1. Evaluation of the Classification Accuracy of Ground Wires and Conductor Points

The manually classified ground wire points and conductor points are used as the ground truth, and the precision-recall method [61] is used to quantitatively evaluate the effectiveness and accuracy of point cloud classification. These are defined as follows:

(13) $$\begin{array}{l} \mathrm{Precision}=\frac{TP}{TP+FP}\\ \mathrm{Recall}=\frac{TP}{TP+FN}\\ F_{\mathrm{score}}=\frac{2\times \mathrm{Precision}\times \mathrm{Recall}}{\mathrm{Precision}+\mathrm{Recall}} \end{array}$$

where *TP*, *FP*, and *FN* represent the quantities of true positive, false-positive, and false-negative results relative to the ground truth, respectively. It is clear that higher precision means fewer false positives and higher recall means fewer false negatives. The F_score_ denotes the comprehensive evaluation of recall and precision.

#### 3.2.2. Evaluation of the Spatial Position Accuracy of Conductor 3D Reconstruction

**Figure 17:**
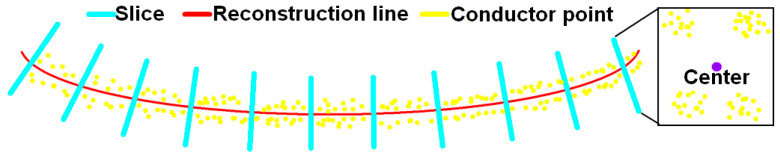
Diagram of slices.

As shown in Figure 17, slices perpendicular to the reconstruction curve with an interval of 1 m are made and the center coordinates of conductor points within each slice are calculated according to Equation (14), which provides the average of the coordinates. Then, perpendicular distances from the center position to the reconstruction curve are computed. To better analyze the error, the root-mean-squared error (RMSE), maximum error (E_max_), and minimum error (E_min_) are used as indicators to measure the position accuracy of the reconstruction result.

(14) $$\left\{ \begin{array}{l} x_{\mathrm{center}}=\frac{\sum_{q=1}^{n}x_{q}}{n}\\ y_{\mathrm{center}}=\frac{\sum_{q=1}^{n}y_{q}}{n}\\ z_{\mathrm{center}}=\frac{\sum_{q=1}^{n}z_{q}}{n} \end{array}\right.$$

Here, ($x_{q},y_{q},z_{q}$) are the coordinates of the conductor points in each slice; *n* is the number of conductor points in each slice.

#### 3.2.3. Evaluation Accuracy of Tree Risk Detection

Based on the point cloud acquired by a terrestrial laser scanner (TLS), the distance between a cluster of tree risk points and conductor points is calculated and the minimum distance is taken as the reference value (RV). Using this method, a large number of reference values can be obtained easily. Equation (15) can be used to calculate the mean absolute error (MAE) between the reference value and the safety distance, which is used to evaluate the accuracy of tree risk point detection.

(15) $$\mathrm{MAE}=\frac{\sum_{i=1}^{m}abs\left(d_{\mathrm{ptp}}^{i}-d_{\mathrm{rv}}^{i}\right)}{m}$$

Here, $d_{\mathrm{ptp}}^{i}$ represents the *i*th safety distance calculated using the method proposed; $d_{\mathrm{rv}}^{i}$ represents the *i*th reference value; and *m* is the minimum distance.

### 3.3. Experimental Results

In this section, the point clouds of transmission lines with different voltage levels are used to conduct experiments and the reliability and practicability of the method proposed in this paper are analyzed according to the experimental accuracy and effect.

#### 3.3.1. The Classification Effect and Accuracy of Ground Wires and Conductor Points

**Figure 18:**
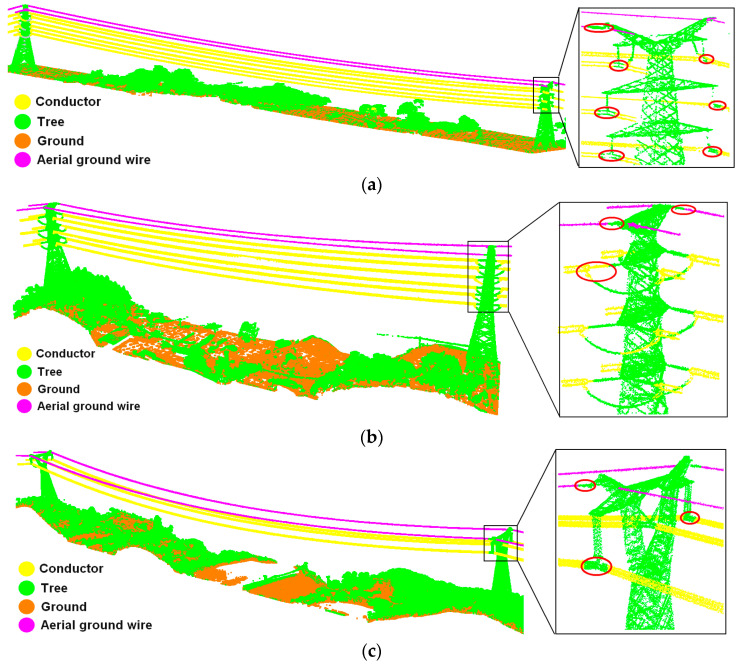
Extraction results for ground wires and conductors: (**a**) classification effect of a ground wire and conductor of a 110 kV transmission line; (**b**) classification effect of a ground wire and conductor of a 220 kV transmission line; (**c**) classification effect of a ground wire and conductor of a 500 kV transmission line. The points in the red circles are misclassified.

As shown in Figure 18, the overall classification effect of the three groups of experimental data is quite good. Considering both conductors and ground wires as the power line category, the main body of the power line is extracted completely, and there is no interruption or misclassification in the middle part of the transmission line. However, the extraction effect of the power line points around the suspending point of the pylon is not good, and a certain proportion of misclassification exists in three groups of data.

Specifically, power lines suspended from the crossing tower are almost perpendicular to the insulator, so the insulator points affect the dimensional feature calculation, resulting in the one-dimensional linear probability of the power line point cloud in this location not being the highest, and the missed extraction of power line points (Figure 18a,c). Power lines suspended from the tensioning tower are almost in line with the insulator and there are drainage lines at the connection, so the one-dimensional linear probability of insulator points and drainage line points at the connection is high, leading to the incorrect extraction of power lines (Figure 18b).

Discussing conductors and ground wires as two categories, the degree of differentiation between the two is clear; there is almost no misclassification of conductor points into ground wire points, so the classification accuracy of both is almost identical. As shown in Table 4, the precision, recall, and F-score of conductor classification exceed 98.05%, 99.00%, and 98.58%, respectively. In addition, Table 5 illustrates the precision, recall, and F-score of ground wire classification accuracy, which are better than 97.98%, 99.14%, and 98.56%, respectively. The above statistics show that the power line extraction method and distinction method between conductors and ground wires proposed in this paper work well, and are suitable for the point cloud classification of different voltage levels. Due to pylon points affecting the dimensional feature calculation of power line points, false positives and false negatives mainly occur at the junction of the power lines and pylons. Furthermore, the structure and shape of the tensioning tower is more complex than the crossing tower, which has a greater negative impact on the dimensional feature calculation, so the power line extraction accuracy for 220 kV is slightly lower than the accuracy for other experimental data.

#### 3.3.2. Spatial Position Accuracy of Conductor 3D Reconstruction

**Figure 19:**
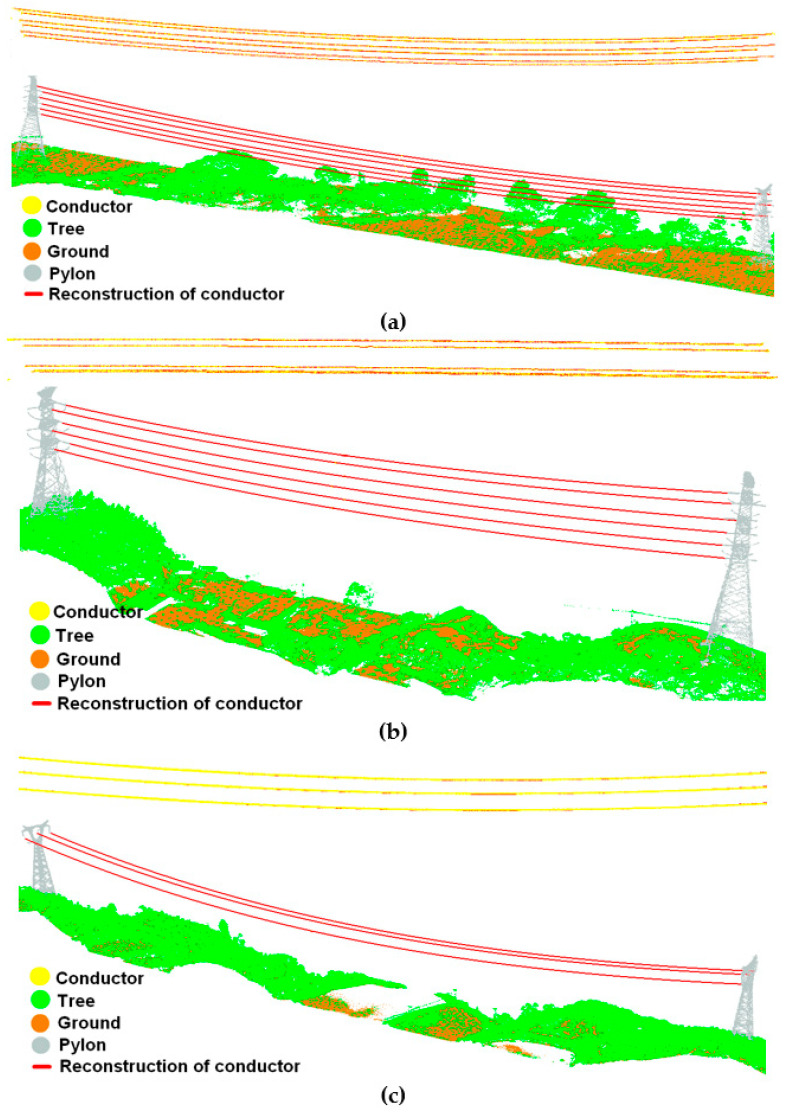
Reconstruction effect of conductors: (**a**) reconstruction of conductors of 110 kV transmission line and matching effect of reconstruction curve and conductor points; (**b**) reconstruction of conductors of 220 kV transmission line and matching effect of reconstruction curve and conductor points; (**c**) reconstruction of conductors of 500 kV transmission line and matching effect of reconstruction curve and conductor points.

As can be seen from Figure 19, the reconstruction results for the conductor are in the center of the conductor points and have a high degree of matching with the conductor points, and the two endpoints of the reconstruction result are closely connected with the suspending points of pylons.

Table 6 shows the RMSE, E_max_, and E_min_ of the distance error between the center coordinates of the conductor points within the slice to the reconstruction curves. From the perspective of horizontal comparison, the RMSE, E_max_, and E_min_ of the 110 kV transmission line are 2.76 cm, 5.34 cm, and 1.91 cm, respectively; the RMSE, E_max_, and E_min_ of the 220 kV transmission line are 3.35 cm, 6.52 cm, and 1.74 cm, respectively; the RMSE, E_max_, and E_min_ of the 500 kV transmission line are 3.67 cm, 7.13 cm, and 2.64 cm, respectively. The maximum errors are all less than twice the RMSE, indicating the stability of the method in this paper. From the perspective of vertical comparison, the RMSE, E_max_, and E_min_ are less than 3.67 cm, 7.13 cm, and 2.64 cm, respectively, which suggests the high accuracy of the method in this paper. The conductor reconstruction accuracy is slightly reduced due to the fact that a higher voltage level means a larger distance between the multi-split conductors contained in a single conductor, but the accuracy is still far better than the error threshold of 0.5 m regulated by the State Grid Corporation of China.

#### 3.3.3. The Accuracy of Tree Risk Detection

**Figure 20:**
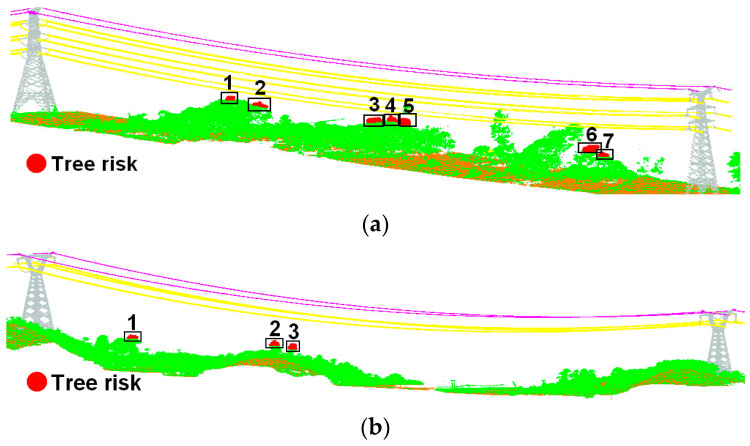
Detection of tree risk points: (**a**) tree risk points of 110 kV transmission lines; (**b**) tree risk points of 500 kV transmission lines. The rectangles show the locations of the tree risk points and the numbers represent the counts of tree risk.

The quantity of tree risk points, efficiency of tree risk detection, and MAE between the reference value and the safety distance calculated using the method in this paper are shown in Table 7. In the three groups of experimental data, the transmission line of 220 kV does not contain tree risk points, which is consistent with the actual situation. However, there are seven and three tree risk points in the transmission line corridors for 110 kV and 500 kV, respectively, as shown in Figure 20. The RV number is consistent with the number of tree risk points detected using the methods in this paper, which indicates the validity of the proposed method. The MAEs of the 110 kV transmission line and 500 kV transmission line are 6.47 cm and 5.53 cm, respectively. Considering that the ranging error of the airborne LiDAR scanner is about 1–2 cm, the true MAE should be less than 5 cm, which is enough to prove that the tree risk point detection method proposed in this paper has high accuracy and meets the transmission line inspection requirements. All experiments were conducted on the same computer with an NVIDIA discrete graphics card, 128 G of RAM, and an Intel i9–10900X CPU. As can be seen from Table 7, the proposed method can effectively reduce the detection time of tree risk points. Due to the long length of transmission lines in China, this method can improve the inspection efficiency while ensuring the correctness of the risk points.

## 4. Conclusions

In this paper, we proposed a power line extraction and tree risk point detection method based on airborne LiDAR for transmission line corridor inspection. Three different voltage levels of transmission line point clouds were used for accuracy and validation verification, and the qualitative analysis showed that the precision, recall, and F-score for conductor classification exceeded 98.05%, 99.00%, and 98.58%, respectively. The precision, recall, and F-score for ground wire classification were better than 97.98%, 99.14%, and 98.56%, respectively, which suggests the high accuracy and good stability of the power line extraction method proposed in this paper. The RMSE, E_max_, and E_min_ of conductor reconstruction were better than 3.67 cm, 7.13 cm, and 2.64 cm, respectively, and the MAE of the safety distance calculation was better than 6.47 cm, proving that the risk point detection method can effectively exclude the interference of non-risk points, quickly clarify the scope of tree risk points, and accurately calculate the clearance distance between conductors and trees, which is significant for the protection of public forestry utilities and the safe operation of public power utilities.

We focused on the detection of tree risk points under realistic working conditions, but no further research has been conducted on the detection methods of tree risk points under various simulated working conditions. In future research, various morphologies and locations of conductors can be simulated by combining the conductor fitting curve and external conditions such as wind, temperature, and ice cover to realize the detection of tree risk points under simulated working conditions. In this process, it will also be necessary to make full use of point cloud intensity information and return numbers to improve the classification effect.

## Figures and Tables

**Table 1:** Dimensional feature probability and information entropy of point under different radii.

Radius	Information Entropy	Probability of Being One-Dimensional	Probability of Being Two-Dimensional	Probability of Being Three-Dimensional
0.1 m	1.108	0.157	0.441	0.402
0.2 m	0.803	0.673	0.256	0.071
0.3 m	0.357	0.823	0.119	0.058
0.4 m	0.163	0.966	0.029	0.005
0.5 m	0.565	0.177	0.021	0.802
0.6 m	0.537	0.149	0.026	0.825
0.7 m	0.507	0.025	0.134	0.841

**Table 2:** Angle statistics between the main eigenvector and the horizontal plane for different classifications of points.

Classification	Count	SD	Maximum	Minimum
Power line	5000	0.235°	4.393°	0°
Pylon	5000	13.061°	32.415°	12.337°
Vegetation	5000	18.172°	43.994°	14.042°

**Table 3:** Detailed information regarding experimental datasets.

Voltage Level	Terrain	Distance	Number of Conductors
110 kV	flat	376 m	6
220 kV	hill	227 m	6
500 kV	mountain	591 m	3

**Table 4:** Classification accuracy of conductor points.

	110 kV	220 kV	500 kV
TP	63,587	89,345	159,859
FN	638	785	1076
FP	1009	1780	2347
Precision	0.9844	0.9805	0.9855
Recall	0.9900	0.9912	0.9933
F-score	0.9880	0.9858	0.9894

**Table 5:** Classification accuracy of ground wire points.

	110 kV	220 kV	500 kV
TP	18,521	15,793	21,776
FN	138	137	142
FP	309	326	334
Precision	0.9836	0.9798	0.9849
Recall	0.9926	0.9914	0.9935
F-score	0.9881	0.9856	0.9892

**Table 6:** Spatial position accuracy of conductor 3D reconstruction.

Voltage Level	RMSE/cm	E_max_/cm	E_min_/cm
110 kV	2.76	5.34	1.91
220 kV	3.35	6.52	1.74
500 kV	3.67	7.13	2.64

**Table 7:** Accuracy of tree risk point detection.

Voltage Level	Consistency of Quantity	Quantities of RVs	Quantities of Tree Risk Points Detected Using Proposed Method	MAE/cm	Efficiency (Point to Point)	Efficiency (Proposed Method)
110 kV	**✓**	7	7	6.47	1.769 s	0.663 s
220 kV	0	0	0	0	1.827 s	0.729 s
500 kV	**✓**	3	3	5.53	2.003 s	0.804 s

## Data Availability

The data used to support the findings of this study are available from the corresponding author upon request.
